# A Meta-Analysis Comparing Different Oral Anticoagulation for the Treatment of Ventricular Thrombus

**DOI:** 10.31083/j.rcm2307243

**Published:** 2022-06-27

**Authors:** Qing Yang, Liyun He, Xin Quan, Yan Liang

**Affiliations:** ^1^National Clinical Research Center of Cardiovascular Diseases, Fuwai Hospital, National Center for Cardiovascular Diseases, Chinese Academy of Medical Sciences and Peking Union Medical College, 100037 Beijing, China; ^2^Emergency Center, Fuwai Hospital, National Center for Cardiovascular Diseases, Chinese Academy of Medical Sciences and Peking Union Medical College, 100037 Beijing, China; ^3^Department of Endocrinology, Key Laboratory of Endocrinology of the National Health Commission, Peking Union Medical College Hospital, Chinese Academy of Medical Sciences and Peking Union Medical College, 100037 Beijing, China; ^4^Department of Echocardiographic, Fuwai Hospital, National Center for Cardiovascular Diseases, Chinese Academy of Medical Sciences and Peking Union Medical College, 100037 Beijing, China

**Keywords:** ventricular thrombus, non-vitamin K antagonists, oral anticoagulants, warfarin

## Abstract

**Background:**

Patients with ventricular thrombus (VT) require anticoagulation therapy and it remains unknown that whether non-vitamin K antagonist oral anticoagulants (NOACs) or vitamin K antagonists (VKAs) are more effective.

**Objective:**

We aimed to compare the effectiveness and safety of NOACs with VKAs on the rate of thrombus resolution and clinical outcomes.

**Methods:**

MEDLINE, PUBMED, EMBASE, Cochrane Library, Web of Science, China National Knowledge Infrastructure Database and Wanfang Database, were searched up to November 22, 2021. The primary outcome was the rate of thrombus resolution, and the secondary outcomes were bleeding, stroke or systemic embolism (SSE), stroke and all-cause death. Odds ratio (OR) and 95% confidential intervals (CI) were used for the pooled results.

**Results:**

Eighteen studies with 1755 participants (NOACs, n = 607; VKAs, n = 1148) were included. There were no significant differences in thrombus resolution (OR 0.92, 95% CI 0.68–1.23, *p *= 0.558), bleeding (OR 0.85, 95% CI 0.54–1.35, *p *= 0.496), SSE (OR 0.77, 95% CI 0.41–1.43, *p *= 0.401), stroke (OR 0.65, 95% CI 0.29–1.49, *p *= 0.312) or all-cause death (OR 1.02, 95% CI 0.63–1.67, *p *= 0.925) between NOACs and VKAs. Subgroup analyses showed a statistics difference in thrombus resolution between NOACs and VKAs among studies which enrolled patients with or without dabigatran (Yes: OR 0.80, 95% CI 0.59–1.08; No: OR 1.48, 95% CI 1.00–2.19; *p* = 0.01), while no significances were observed according to baseline characteristics.

**Conclusions:**

Our findings showed that NOACs were comparable to VKAs in thrombus resolution as well as clinical outcomes. In studies that enrolled patients without dabigatran, the thrombus resolution seemed to be greater in NOACs group than VKAs group. And in different proportion of baseline left ventricular ejection fraction, history of ischemic cardiomyopathy and combination with antiplatelet, the thrombus resolution among the two groups remained similar.

## 1. Introduction

Ventricular thrombus (VT), with an incidence ranging from 2% to 5% [1, 2], can lead to a high rate of embolism to vital organs or mortality [3, 4, 5], which are mostly secondary to severe cardiac systolic dysfunction, myocardial infarction or cardiomyopathy. Treatments and clinical outcomes in patients with VT were inconsistent. According to guidelines, the warfarin use was reasonable for ST elevation myocardial infarction (STEMI) patients with asymptomatic left VT (*Class II a, Level C evidence*) [6, 7]. Due to the inherent limitations of warfarin, patients might have a poor compliance, making it arduous to guarantee the effective maintenance and control of the therapeutic target of international normalized ratio (INR).

Non-vitamin K antagonist oral anticoagulants (NOACs) have been an attractive anticoagulant choice nowadays in the setting of non-valvular atrial fibrillation and venous thrombotic diseases [8, 9]. Several studies reported that patients with VT who received NOACs had a great rate of thrombus resolution (83% to 100%) [10, 11]. In general, compared with vitamin K antagonists (VKAs), NOACs have several special features such as a rapid onset of action, offering a more predictable and flexible anticoagulant option, and have been increasingly favored in clinical practice [12]. In the guideline of stroke, patients with acute myocardial infarction (AMI) combined with ischemic stroke or transient ischemic attack (TIA) were recommended to use dabigatran, rivaroxaban or apixaban for 3 months to prevent recurrence stroke or TIA (*Class II b, Level C evidence*) [13]. Similarly, 2017 European guideline suggested that STEMI patients with left VT should maintain anticoagulation therapy for up to 6 months under the guidance of repeated imaging (*Class II a, level C evidence*) [14]. Up to date, the application of NOACs in patients with VT has not been clearly evaluated and the comparison between NOACs and VKAs in patients with VT remains controversial.

To address the knowledge gaps, we aimed to conduct a systematic review and meta-analysis to compare the effectiveness and safety between NOACs and VKAs, providing more evidence on anticoagulation therapy for patients with VT.

## 2. Methods

The study was performed under the guidelines of the Preferred Reporting Items for Systematic reviews and Meta-Analyses (PRISMA) statement [15] and it was registered on PROSPERO as CRD42020205477, which was available from: https://www.crd.york.ac.uk/prospero/#recordDetails.

### 2.1 Search Strategy

Two reviewers (Q.Y. and L-Y.H.) independently searched seven databases including Cochrane Library, MEDLINE (Ovid), PUBMED, EMBASE, Web of Science, China National Knowledge Infrastructure (CNKI) Database and Wanfang Database to identify the studies from inception to November 22, 2021. The reference lists of researches and systematic reviews were also reviewed and retrieved for more trials. Potential gray literature was searched in OpenGrey.eu. The following search terms were used: “ventricular thrombus” or “intraventricular thrombus”, “direct/new/novel oral anticoagulants” or “non-vitamin K antagonists oral anticoagulants”, “warfarin”, “vitamin K antagonists” and combination of these terms as keywords (The detailed search strategy was showed in **Supplementary materials**).

### 2.2 Study Eligibility and Selection

Eligible studies met all the following criteria: (1) studies that included participants with VT, regardless of nationality, sex, race, occupation or education; (2) studies that compared NOACs and VKAs for the treatment of VT (NOACs could be rivaroxaban, apixaban, edoxaban, dabigatran, or any other new oral anticoagulants and VKAs could be warfarin, coumadin, phenprocoumon, acenocumarol, fluindione, phenindione or anisindione).

Studies that met the following criteria were excluded: (1) studies that were abstracts, reviews, duplicated publications, case reports or case series; (2) the data were incomplete or not serious, especially the important outcome events missing or not available.

Investigators searched for related literature, imported articles into the database created by Endnote (Endnote X9.3.1; Thomson Reuters, San Francisco, CA), and filtered for duplicates. According to the inclusion and exclusion criteria, our two reviewers (Q.Y. and L-Y.H.) independently screened the titles and abstracts. If appropriate, full texts of the records were reviewed to identify all potentially eligible studies. The selection processes were repeated twice. Conflicts were resolved through discussions with other team members until a consensus was reached.

### 2.3 Data Collection and Analysis

Data collection was conducted to extract the participant and study characteristics, including study design, baseline information of subjects (age, sex, hypertension, diabetes mellitus, atrial fibrillation, history of thromboembolism, ischemic cardiomyopathy (ICM), dyslipidemia), cardiac imaging data (left ventricular ejection fraction, LVEF), and antiplatelet therapy.

The primary outcome was the rate of thrombus resolution which was confirmed by echocardiogram, computer tomography (CT) or cardiac magnetic resonance imaging (CMR), and the secondary outcomes included bleeding, stroke, stroke or systemic embolism (SSE) events and all-cause death. Stroke events referred to definite ischemic or hemorrhagic stroke, and other uncertain or unknown stroke [16]. SSE events were defined as the stroke or transient ischemic attack, acute coronary emboli (including myocardial infarction) or acute peripheral artery emboli (limb, renal, or digestive arteries) [17]. Bleeding events were defined as International Society on Thrombosis and Haemostasis (ISTH) major bleeding or clinically relevant non-major bleeding events [18, 19]. Two reviewers (Q.Y. and L-Y.H.) extracted the data independently and compared the results to ensure coherence, and an additional reviewer resolved the discrepancies.

### 2.4 Assessment of Quality in the Included Studies

The Newcastle-Ottawa Scale (NOS) was operated by two reviewers independently to evaluate the quality of studies included [20], which assessed cohort studies for three blocks, including selection, comparability, and outcome evaluation. A study could be awarded a maximum of one star for each numbered item within the selection and outcome categories. A maximum of two stars could be given for comparability. The article quality was evaluated as follows: low quality (0–3); moderate quality (4–6); high quality (7–9). Any disagreement was discussed with other team members until agreement was reached.

### 2.5 Statistics Analysis

The continuous data were presented as mean and standard deviation (SD) or median and interquartile range (IQR), and the dichotomous outcomes were calculated by the odds ratio (OR) with 95% confidence intervals (CIs) [21]. A random-effects model was used for meta-analysis considering the possible heterogeneity existing in the eligible studies. Heterogeneity was visually assessed with the forest plots and statistically detected by standard Chi-squared test and *$I^{2}$* statistic [22]. *$I^{2}$* test explained the percentage of variation in intervention estimates due to heterogeneity rather than sampling error, with *$I^{2}$* values 0% to 40% being indicative of likely insignificant; 30% to 60%, likely moderate heterogeneity; 50% to 90%, likely substantial heterogeneity; 75% to 100%, substantial heterogeneity [21]. To explore the possible sources of heterogeneity in the thrombus resolution, subgroup analyses were performed (a. Studies with dabigatran vs. studies without dabigatran; b. ICM history ≥80% vs. <80%; c. LVEF ≥30% vs. <30%; d. Antiplatelet therapy ≥90% vs. <90%) according to the baseline characteristics that might be related to the rate of thrombus resolution. The generalized linear model (generalized linear mixed-model, GLMM) was conducted to reduce the bias of classical Meta-analysis caused by continuous correction. Sensitivity analysis was performed by omitting studies one by one. Publication bias was visually evaluated using funnel plot and statistically accessed by Egger’s regression tests [23]. Furthermore, when Egger’s regression tests or funnel plots indicated publication bias, we utilized the trim-and-fill method to identify whether funnel plot asymmetry should be corrected [24]. All comparisons were considered two-sided, and the* p *< 0.05 was considered as statistical significance. All the analyses were scheduled for completion with R Studio, Version 3.5.1 (R Foundation for Statistical Computing, Vienna, Austria).

## 3. Results

### 3.1 Literature Search

**Fig. 1.**
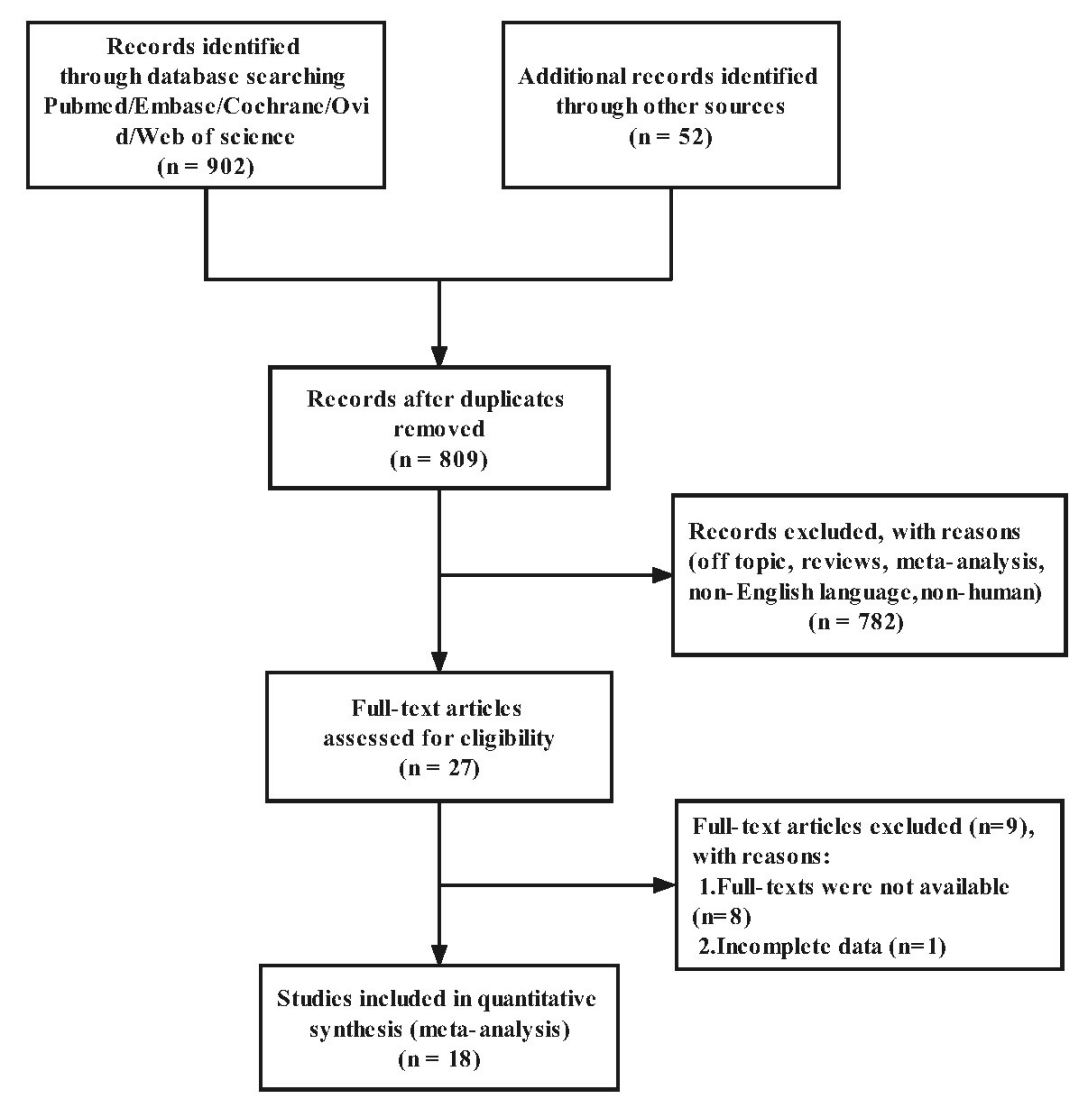
**Flow chart of literature search strategies**.

Fig. 1 represented the process of the literature search. A total of 954 citations were yielded by searching Cochrane Library, MEDLINE, PUBMED, EMBASE, and Web of Science, CNKI and Wanfang Database, of which 809 records remained after removing the duplicates. After reviewing titles and abstracts, 27 citations were remained for the full-text screening and 782 records were deleted owing to irrelevance to our study. Nine articles were deleted due to the inaccessibility of full-text (n = 8) and uncomplete data (n = 1). Finally, we included a total of 18 eligible studies for meta-analysis (17 for retrospective study and one for prospective study) [25, 26, 27, 28, 29, 30, 31, 32, 33, 34, 35, 36, 37, 38, 39, 40, 41, 42].

### 3.2 Study and Patient Characteristics

Among the eligible studies included, eight studies were conducted in 2021, and the rest were published in recent years. Observational durations ranged from 3 to 172 months, eight of which lasted for 12 months or even longer [27, 28, 29, 38, 40, 41] (Table 1, Ref. [25, 26, 27, 28, 29, 30, 31, 32, 33, 34, 35, 36, 37, 38, 39, 40, 41, 42]). Among 1755 patients included, 65% of patients (n = 1148) received VKAs and 35% of patients (n = 607) received NOACs. Rivaroxaban (70.4%, 405/575) was the most commonly used NOACs, followed by apixaban (24.0%, 138/575), dabigatran (5.4%, 31/575), and edoxaban (0.2%, 2/575).

**Table 1.** **Baseline characteristics of studies included**.

Study	Follow-up, months	Comparison	Sample size	Male, n (%)	Mean age, years	Mean LVEF, %	TTR, %	Antiplatelet therapy, n (%)	Major medical history, n (%)		
									Atrial fibrillation	Thromboembolisms	Ischemic cardiomyopathy
Daher *et al*., 2020 [25]	3	NOACs	17	14 (82.4)	57	41	-	11 (64.7)	-	-	15 (88)
		VKA	42	35 (83)	61	36		28 (67)	-	-	36 (74)
Iqbal *et al*., 2020 [26]	36	NOACs	22	16 (73)	62	31	-	9 (41)	3 (14)	2 (9)	18 (82)
		VKA	62	57 (92)	62	35		39 (65)	3 (5)	11 (17.7)	55 (89)
Robinson *et al*., 2020 [27]	12	NOACs	121	94 (77.7)	58.1	27.7	-	77 (63.6)	30 (24.8)	79 (65.3)	66 (54.5)
		VKA	236	170 (72)	58.2	28.2		164 (69.5)	45 (19.1)	123 (52.1)	148 (62.7)
Jones *et al*., 2020 [28]	24	NOACs	41	33 (80.4)	58.7	33.5	53.5	38 (92.7)	0	21 (55.3)	21 (55.3)
		VKA	60	51 (85)	60.8	35.4		55 (91.7)	0	22 (36.7)	22 (36.7)
Guddeti *et al*., 2020 [29]	12	NOACs	19	15 (79)	60.7	25	-	11 (57.9)	4 (21.1)	11 (57.9)	10 (52.6)
		VKA	80	55 (68.8)	61.3	25		54 (67.5)	18 (22.5)	49 (61.2)	48 (60)
Yan *et al*., 2019 [30]	6	NOACs	11	9 (81.8)	64.2	38.6	-	11 (100)	0	-	11 (100)
		VKA	37	34 (91.9)	59	39.6		21 (56.7)	0	-	37 (100)
Chao *et al*., 2018 [32]	8.3	NOACs	56	45 (80.3)	61.2	46.8	-	56 (100)	4 (7.1)	50 (89.3)	47 (83.9)
		VKA	70	55 (78.6)	60.4	35.3		70 (100)	6 (8.6)	67 (95.7)	61 (87.1)
Li *et al*., 2015 [31]	3	NOACs	15	11 (73.3)	51.6	-	-	0	5 (33)	6 (40)	6 (40)
		VKA	16	12 (75)	52.4	-		0	4 (25)	7 (43)	7 (43)
Zhang *et al*., 2021 [38]	24	NOACs	33	24 (72.7)	60.3	42.9	77.4	33 (100)	-	-	33 (100)
		VKA	31	23 (74.2)	61.3	41.4		31 (100)	-	-	31 (100)
Mihm *et al*., 2021 [34]	6	NOACs	33	23 (69.7)	63.3	32.58	-	19 (57.6)	13 (39.4)	9 (27.3)	14 (42.4)
		VKA	75	54 (72)	60.3	27.95		55 (73.3)	15 (20)	20 (26.7)	72 (96)
Alcalai *et al*., 2021 [36]	3	NOACs	18	13 (72.2)	55.5	35	60	18 (100)	-	-	4 (22.2)
		VKA	17	15 (88.2)	58.8	36		17 (100)	-	-	3 (17.7)
Albabtain *et al*., 2021 [35]	9.5	NOACs	28	24 (85.7)	58.2	26.4	-	19 (67.9)	1 (3.6)	16 (57.1)	16 (57.1)
		VKA	35	34 (97.1)	59	27.3		20 (57.1)	2 (5.7)	25 (71.4)	25 (71.4)
Yao *et al*., 2021 [42]	3	NOACs	42	34 (81)	58.5	42.8	-	-	-	-	25 (59.5)
		VKA	58	48 (82.8)	60.1	36.6		-	-	-	32 (55.2)
Willeford *et al*., 2020 [40]	172.5	NOACs	22	17 (77.3)	54	-	-	5 (22.7)	3 (13.6)	6	15 (68.2)
		VKA	129	104 (80.6)	56	-		70 (54.3)	24 (18.6)	46	68 (52.7)
Iskaros *et al*., 2021 [33]	3	NOACs	32	28 (88)	62	25	-	21 (66)	4 (13)	20	22 (69)
		VKA	45	41 (91)	63	25		30 (67)	9 (20)	25	33 (73)
Varwani *et al*., 2021 [39]	12	NOACs	58	-	-	-	13.1	-	-	-	-
		VKA	34	-	-	-		-	-	-	-
Cochran *et al*., 2020 [37]	12	NOACs	14	11 (78.6)	51.5	-	-	-	-	-	26 (53)
		VKA	59	45 (76.3)	62	-		-	-	-	36 (61)
Xu *et al*., 2021 [41]	2.4	NOACs	25	19 (76)	59.4	33.8	-	11 (44)	20 (80)	4 (16)	18 (72)
		VKA	62	47 (75.8)	61.9	37.6		27 (43.5)	50 (80.6)	13 (21)	48 (77.4)

Abbreviations: NOACs, non-vitamin K antagonist oral anticoagulants; VKAs, vitamin K antagonists; LVEF, left ventricular ejection fraction.

Thirteen studies adopted echocardiogram to assess VT [25, 27, 29, 31, 32], three studies using echocardiogram or CMR [14, 28, 34] and two studies adopted all three tools-echocardiogram, CT, CMR-to confirm thrombus resolution [30, 33]. Most of the studies had a higher proportion of men than women. Among the total of 1755 patients, the mean age of participants varied from 51 to 64 years, with a median age of 60.3 years old. Among the included studies, four papers reported time in therapeutic range (TTR) [28, 36, 38, 39], of which two articles indicated that patients met the therapeutic target of INR (TTR ≥60%) [36, 38], while the rest of fourteen articles reported no tracked data of TTR even though they collected the baseline of INR or highlighted the importance of monitoring the INR range of 2.0–3.0. Fourteen studies reported baseline LVEF which ranged from 25% to 46%, and patients in NOACs group had a median of 33.6% (IQR 28.5%–44.4%) while patients in VKAs group had a median of 35.3% (IQR 28.0%–36.4%). Fifteen studies reported the combination of antiplatelet agents at baseline, with 68.8% (339/493) patients in NOACs group and 68.3% (681/997) in VKAs group (Table 1).

### 3.3 Quality Assessment

The quality assessment showed that the quality of 11 studies was at high levels while the rest was at moderate levels, and the average score was 7.11 (**Supplementary Table 1**). All studies had adequacy of follow-up by a description of missing visits. Owing to the retrospective studies, all of them had record linkages. Five studies did not adequately consider the comparability of the exposed and unexposed groups in their design and statistical analysis [25, 29, 30, 31, 32].

### 3.4 Outcomes of Meta-Analysis

#### 3.4.1 Thrombus Resolution

**Fig. 2.**
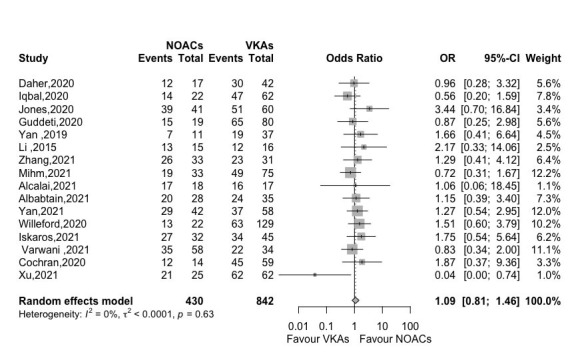
**Forest plot of thrombus resolution between NOACs versus VKAs (16 studies)**. Abbreviation: OR, odds ratio; CI, Confidence interval; NOACs, non-vitamin K antagonist oral anticoagulants; VKAs, vitamin K antagonists.

At a median follow-up period of 8.9 (IQR 3–12) months, 71% (599/842) of patients in the VKAs group and 74% (319/430) in the NOACs group had complete thrombus resolution. Fig. 2 showed no significant difference in thrombus resolution rate between NOACs and VKAs groups (OR 1.09, 95% CI 0.81–1.46, *p *= 0.558) with insignificant heterogeneity (*$I^{2}$* = 0%) by analyzing 15 retrospective studies and 1 prospective study (Fig. 2).

#### 3.4.2 Bleeding

**Fig. 3.**
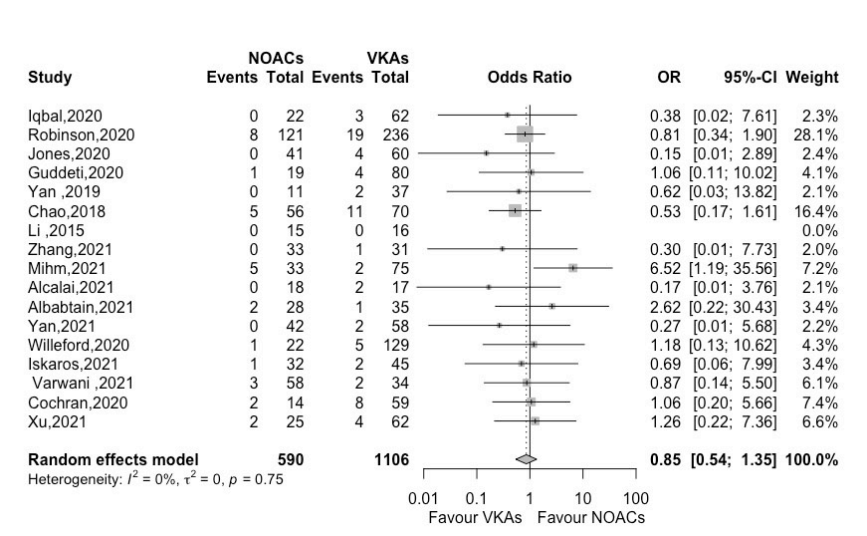
**Forest plot of bleeding between NOACs versus VKAs (17 studies)**. Abbreviation: OR, odds ratio; CI, Confidence interval; NOACs, non-vitamin K antagonist oral anticoagulants; VKAs, vitamin K antagonists.

No significant difference was observed in bleeding rate between NOACs and VKAs groups (OR 0.85, 95% CI 0.54–1.35, *p *= 0.496, *$I^{2}$* = 0%) according to 17 studies (Fig. 3).

#### 3.4.3 Stroke or Systemic Embolism

**Fig. 4.**
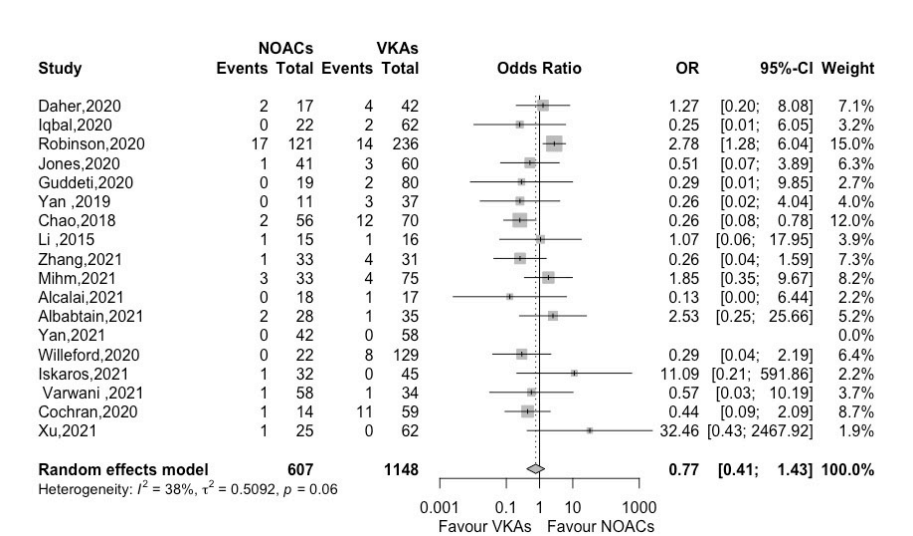
**Forest plot of SSE between NOACs versus VKAs (18 studies)**. Abbreviation: OR, odds ratio; CI, Confidence interval; NOACs, non-vitamin K antagonist oral anticoagulants; VKAs, vitamin K antagonists.

A total of 18 studies reported the outcome of SSE and no significant difference was found in the comparison of NOACs and VKAs groups (OR 0.77, 95% CI 0.41–1.43, *p *= 0.401) with moderate heterogeneity (*$I^{2}$* = 38%) (Fig. 4).

#### 3.4.4 Stroke

**Fig. 5.**
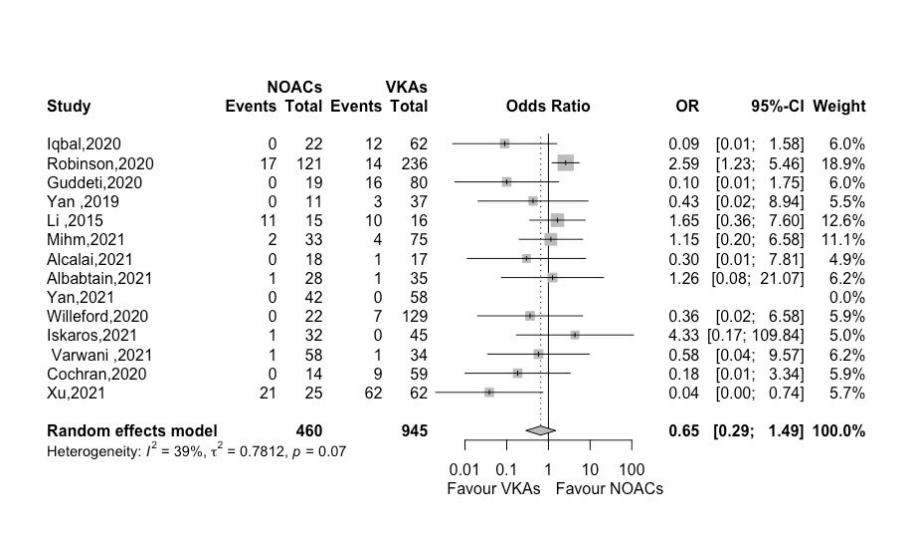
**Forest plot of stroke between NOACs versus VKAs (14 studies)**. Abbreviation: OR, odds ratio; CI, Confidence interval; NOACs, non-vitamin K antagonist oral anticoagulants; VKAs, vitamin K antagonists.

Apart from analyzing SSE in our included studies, we also extracted the stroke events in 14 studies and there was no significant difference as well (OR 0.65, 95% CI 0.29–1.49, *p *= 0.312, *$I^{2}$* = 39%) (Fig. 5).

#### 3.4.5 All-Cause Death

**Fig. 6.**
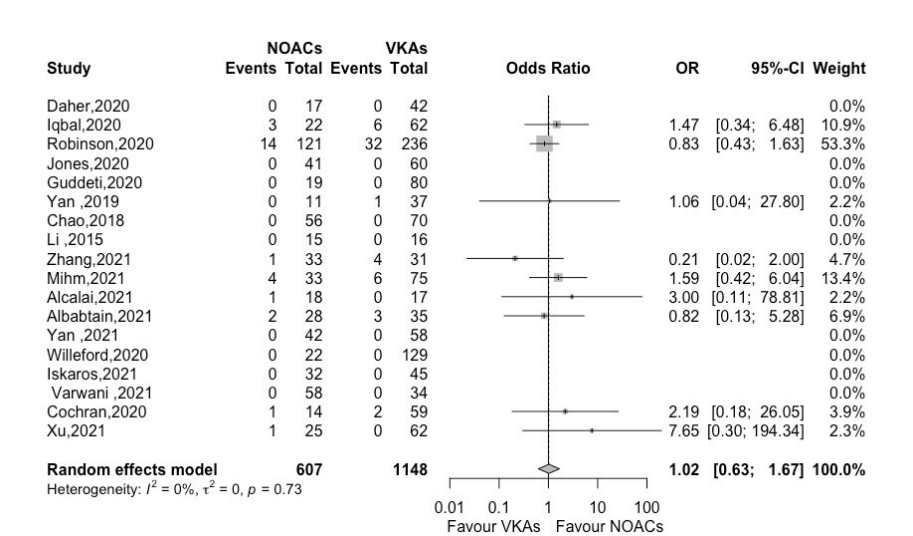
**Forest plot of all-cause death between NOACs versus VKAs (18 studies)**. Abbreviation: OR, odds ratio; CI, Confidence interval; NOACs, non-vitamin K antagonist oral anticoagulants; VKAs, vitamin K antagonists.

Fig. 6 graphed that an insignificant difference was observed in all-cause death between NOACs and VKAs groups (OR 1.02, 95% CI 0.63–1.67, *p *= 0.925) with small heterogeneity (*$I^{2}$* = 0%) in 18 studies.

**Supplementary Table 2** summarized the results for efficacy and safety of NOACs versus VKAs. In order to add the double zero events of outcomes into the analysis, the GLMM was performed to give unbiased results. **Supplementary Figs. 1–5** showed the results of thrombus resolution, bleeding, stroke, SSE and all-cause death based on the GLMM, and no significances were observed.

### 3.5 Subgroup Analysis

Table 2 and **Supplementary Figs. 6–9** showed the subgroup analyses on the thrombus resolution according to several interesting baseline characteristics from the clinic knowledge, consensus of experts and international guidelines [6, 7, 13, 14]. Considering the possible differences among the direct thrombin inhibitor dabigatran vs. the other NOACs, we performed the analysis and observed a statistical difference among studies that enrolled patients either with dabigatran or without dabigatran (Yes: OR 0.80, 95% CI 0.59–1.08, *$I^{2}$* = 11%; No: OR 1.48, 95% CI 1.00–2.19, *$I^{2}$* = 0%; *p* = 0.01). In six studies with the median LVEF ≥30%, NOACs group showed a similar thrombus resolution rate (OR 0.96, 95% CI 0.53–1.73, *$I^{2}$* = 41%) compared with VKAs group, and the result remained in the other four studies with a median LVEF <30% (OR 0.86, 95% CI 0.56–1.31, *$I^{2}$* = 0%). And to explore the potential effect of antiplatelet therapy combined with anticoagulation in patients with VT, studies were divided into two groups based on a high or moderate rate of combination of antiplatelet therapy. And when analyzing studies with antiplatelet therapy ≥90%, NOACs had a greater thrombus resolution than VKAs (OR 1.72, 95% CI 0.71–4.19, *$I^{2}$* = 0%) while considering studies with antiplatelet therapy <90%, it came to an opposed result between the two groups (OR 0.86, 95% CI 0.65–1.14, *$I^{2}$* = 9%), though both of which had no statistical significance. Moreover, we conducted a further analysis in different prevalence of ICM history among these included studies, and the result remained non-significant (ICM ≥80%: OR 1.15, 95% CI 0.73–1.81, *$I^{2}$* = 15%; ICM <80%: OR 1.08, 95% CI 0.70–1.65, *$I^{2}$* = 35%; *p* = 0.85). Based on the above information, we could subscribe the possible sources of heterogeneity to the baseline LVEF and the history of ICM for the analysis of thrombus resolution, owing to the moderate high *$I^{2}$*.

**Table 2.** **Subgroup analyses on the thrombus resolution of NOACs versus VKAs**.

Subgroup		K	Pooled OR (95% CI)	Heterogeneity	*p* value*
Dabigatran					0.01
	${\mathrm{Yes}}^{†}$	8	0.80 (0.59–1.08)	11%	
	No	9	1.48 (1.00–2.19)	0%	
Baseline LVEF					0.76
	≥30%	6	0.96 (0.53–1.73)	41%	
	<30%	4	0.86 (0.56–1.31)	0%	
Baseline antiplatelet therapy					0.14
	≥90%	3	1.72 (0.71–4.20)	0%	
	<90%	11	0.86 (0.65–1.14)	9%	
Baseline prevalence ischemic cardiomyopathy					0.85
	≥80%	7	1.15 (0.73–1.81)	15%	
	<80%	9	1.08 (0.70–1.65)	35%	

*Test for subgroup difference (*p *< 0.05 for statistically significant). †Studies in which the NOACs groups use included dabigatran. Abbreviations: K, Number of studies; OR, odds ratio; CI, Confidence interval; NOACs, non-vitamin K antagonist oral anticoagulants; VKAs, vitamin K antagonists; LVEF, ventricular ejection fraction.

### 3.6 Sensitivity Analyses

We conducted sensitivity analyses on the thrombus resolution, SSE, stroke, bleeding and all-cause death, which omitted each study one by one to examine the impacts of any individual study on the final results (**Supplementary Figs. 10–14**). The omission of any individual study did not significantly change the overall results.

### 3.7 Publication Bias

No visible publication bias was found in the study, which was visually exhibited through the trimmed funnel plot (**Supplementary Figs. 15,16**). The effect after shearing was OR 0.84 (95% CI 0.46–1.54) which showed that the mild publication bias had no substantial effect on the overall results. Statistical evaluations via Egger’s test showed no significant publication bias (*p* = 0.601).

## 4. Discussion 

Our systematic review and meta-analysis evaluated the efficacy and safety between NOACs and VKAs in the treatment of VT quantitatively and systematically, which included a large number of full-text prospective and retrospective studies. We found no significant differences in VT resolution, SSE, stroke, bleeding events or all-cause death in the comparison of the two agents, with or without adjusting the confounding.

Researchers have explored the effectiveness of NOACs in the treatment of VT [25, 26, 43]. Tomasoni *et al*. [44] conducted a summary of single-armed case series, including 52 patients with left VT, 93.2% had complete thrombus resolved in a median follow-up of 180 days. No stroke or embolism events were observed, and only one patient experienced nonfatal bleeding. Numerous studies claimed that NOACs were comparable with or even outweighed VKAs [28, 33, 34, 35, 36, 37, 39, 40, 45, 46, 47], and obviously the most popular or dominant usage of NOACs was rivaroxaban in all studies. It was known that NOACs could be divided into two types targeting various action mechanism-anti-Xa inhibitor (rivaroxaban, apixaban and edoxaban or anti-IIa inhibitors (dabigatran). Compared anti-Xa, dabigatran has low bioavailability and is mainly cleared by the kidney, thus patients with moderate renal function should avoid use. In the field of cardiovascular diseases such as atrial fibrillation or pulmonary embolism, NOAC have all been proven at least as safe and effective as warfarin in large randomized controlled trials [8, 48, 49]. And neither guidelines nor studies have identified the differences in the treatment of VT in anti-Xa inhibitors vs. dabigatran, which may be due to the low proportion of patients included in retrospective studies. From our subgroup analysis, in studies that included patients without dabigatran, the thrombus resolution favored NOACs group over VKAs group. Though there was a statistical significance in studies with dabigatran or without dabigatran, the result needed to be interpreted carefully since the sample of patients who were administered with dabigatran was small in our study. To conclude, patients with VT might obtain inconsistent results with the usage of different NOACs.

Several meta-analyses reported similar results to ours [37, 47, 50, 51, 52]. Dalia *et al*. [52] included a total of eight studies and reported non-significant differences in thrombus resolution, stroke or SSE, bleeding and mortality in the comparison of NOACs and VKAs, though three of including papers were conference abstracts. Cocharan *et al*. [37] including six studies also showed that NOACs group was similar to VKAs in the rate of unresolved thrombus, embolic events or bleeding events. Interestingly, Camilli *et al*. [53] found that NOACs had a lower bleeding rate and an increase in SSE events compared with VKAs. Otherwise, one meta-analysis including the same studies as Camilli *et al*. [53] concluded no significant difference in each outcome [47]. Whether NOACs could decrease the risk of bleeding or stroke was unknown. And considering patients who had a great adherence or kept the TTR more than 60% could have a reduced risk of bleeding or stroke events, further large randomized controlled trials are required to assess the net safety efficacy profile of NOACs compared to VKAs different in VKAs recipients.

Our meta-analysis had three advantages. Firstly, the current study merged recent full-text articles from various countries comparing the effectiveness and safety of NOACs and VKAs, including both prospective and retrospective observational studies, which offered a global picture of comparative outcomes with NOACs and VKAs in the rate of VT resolution. Then, in order to explore the possible sources of heterogeneity in the thrombus resolution, we performed subgroup analyses based on clinical variables that might be related to the rate of thrombus resolution, and found that, different NOACs targeting various inhibitors produced inconsistent results in the treatment of VT. More importantly, for minimizing the confounding, we conducted the generalized linear mixed-model to give unbiased estimates in the presence of missing data, providing reasonably stable results.

There were several limitations in our paper. Firstly, only observational studies were included which could not make causal inference, though we conducted subgroups to minimize the difference of patient characteristics within the two groups. However, we would continue to keep track of those upcoming clinical trials to add more robust persuasion. Secondly, the imaging tool for the diagnosis of VT was inconsistent, which decreased the accuracy and precision of the assessment of VT. Another problem was that, given the nature of the meta-analysis, we had no more data on INR to better control the equivalence of the two treatment strategies.

With the increasing use of NOACs in clinical practice, NOACs have been gradually favored by physicians in the treatment of VT, except for patients with antiphospholipid syndrome or severe renal insufficiency. Considering the clinical practicability and health economics, VKAs had numerous inherent disadvantages such as the slow onset of action, susceptibility to food and narrow therapeutic window, which notably lead to a poor treatment compliance, and on the other hand, in the era of COVID-19 pandemic, patients with VT obtained more benefits with the treatment of NOACs since it was so formidable to frequently monitor the INR or contact with clinic workers when administering with VKAs, which echoed the recommendation of Thachil *et al*. [54] and Hermans *et al*. [55]. Therefore, NOACs can be considered a favorable alternative for clinics and patients especially in patients intolerant to VKAs therapy. In addition, NOACs may not only influence the outcome of thrombus resolution, but also have a protective effect against some cardiac diseases. Recently, Jumeau *et al*. [56] found that NOACs could slow down the process of atrial dilation by preventing interstitial fibrosis, extracellular interstitial remodeling, and heart failure-associated atrial hypertrophy, and also improve left ventricular remodeling while reducing left atrial size and left ventricular diameter, the latter of which could further promote thrombus regression.

Overall, it is critical to explore the therapeutic effectiveness and safety of NOACs on VT in large randomized controlled trials, as well as to offer the type, dose or duration of treatment of NOACs. Until now, there are four trials in the pilot phase, comparing NOACs versus warfarin (EARLY-MYO-LVT trial [57], NCT 03415386, NCT02982590), which can provide strong evidence for future work on this topic area.

## 5. Conclusions

Our findings showed that no statistically significant differences were observed in thrombus resolution, SSE, stroke, bleeding events or all-cause death between NOACs and VKAs in patients with VT. In studies that enrolled patients without dabigatran, NOACs group might have a greater thrombus resolution than VKAs group. Further well-designed prospective clinical trials are required to determine the efficacy and safety of the agents.

## Abbreviations

VT, ventricular thrombus; NOACs, non-vitamin K antagonist oral anticoagulants; VKAs, vitamin K antagonists; OR, odds ratio; CI, confidential intervals; SD, standard deviation; AMI, acute myocardial infarction; LVEF, ventricular ejection fraction; ICM, ischemic cardiomyopathy; STEMI, ST elevation myocardial infarction; TIA, transient ischemic attack; PRISMA, Preferred Reporting Items for Systematic reviews and Meta-Analyses; CNKI, China National Knowledge Infrastructure; TTR, time in therapeutic range; INR, international normalized ratio; CT, computer tomography; CMR, cardiac magnetic resonance imaging; ISTH, International Society on Thrombosis and Haemostasis; NOS, Newcastle-Ottawa Scale.

## Supplementary Material

Supplementary material associated with this article can be found, in the online version, at https://doi.org/10.31083/j.rcm2307243.
