# Supplementary material for: A Meta-Analysis Comparing Different Oral Anticoagulation for the Treatment of Ventricular Thrombus
Source: Rev Cardiovasc Med. 2022 Jun 27;23(7):243. doi: 10.31083/j.rcm2307243 (PMC11266787; doi:10.31083/j.rcm2307243)
Supplement: Supplementary file 1 [file 2153-8174-23-7-243-s1.docx]

Supplementary Table 1. Assessment of qualities of the studies included in the meta-analysis.

| Study | Selection | Comparability | Outcome | Total^a^ | Quality^b^ |
| --- | --- | --- | --- | --- | --- |
| Zhang *et al.,* 2021 | 3 | 1 | 3 | 7 | high |
| Mihm *et al.,* 2021 | 4 | 0 | 3 | 7 | high |
| Alcalai *et al.,* 2021 | 3 | 1 | 2 | 6 | moderate |
| Albabtain *et al.,* 2021 | 4 | 1 | 3 | 8 | high |
| Yao *et al.,* 2021 | 3 | 1 | 2 | 6 | moderate |
| Iskaros *et al.,* 2021 | 4 | 1 | 2 | 7 | high |
| Varwani *et al.,* 2021 | 4 | 1 | 3 | 8 | high |
| Xu *et al.,* 2021 | 3 | 1 | 2 | 6 | moderate |
| Willeford *et al.,* 2020 | 4 | 2 | 3 | 9 | high |
| Cochran *et al.,* 2020 | 3 | 1 | 2 | 6 | moderate |
| Daher *et al.,* 2020 | 3 | 1 | 2 | 6 | moderate |
| Iqbal *et al.,* 2020 | 4 | 1 | 3 | 8 | high |
| Robinson *et al.,* 2020 | 4 | 2 | 3 | 9 | high |
| Jones *et al.,* 2020 | 4 | 1 | 3 | 8 | high |
| Guddeti *et al.,* 2020 | 4 | 1 | 3 | 8 | high |
| Yan *et al.,* 2019 | 3 | 1 | 3 | 7 | high |
| Chao *et al.,* 2018 | 3 | 1 | 2 | 6 | moderate |
| Li *et al.,* 2015 | 3 | 1 | 2 | 6 | moderate |

^a^ Sum scores were calculated.

^b^ Low quality = 0-3; moderate quality = 4-6; high quality = 7-9.

Supplementary Table 2. Summary of the results for efficacy and safety of NOACs versus VKAs.

| **Outcomes** | **K** | **Total N** | **Pooled OR**  **(95% CI)** | ***P* value** | **Heterogeneity** | |
| --- | --- | --- | --- | --- | --- | --- |
|  |  |  |  |  | ***I^2^*** | ***P* value** |
| **Thrombus resolution** | |  |  |  |  |  |
| NOACs versus VKAs | 16 | 1272 | 1.09 (0.81–1.46) | 0.558 | 0% | 0.63 |
| **Bleeding** |  |  |  |  |  |  |
| NOACs versus VKAs | 17 | 1696 | 0.85 (0.54–1.35) | 0.496 | 0% | 0.75 |
| **SSE** |  |  |  |  |  |  |
| NOACs versus VKAs | 18 | 1755 | 0.77 (0.41–1.43) | 0.401 | 38% | 0.06 |
| **Stroke** |  |  |  |  |  |  |
| NOACs versus VKAs | 14 | 1405 | 0.65 (0.29–1.49) | 0.312 | 39% | 0.07 |
| **All-cause death** |  |  |  |  |  |  |
| NOACs versus VKAs | 18 | 1755 | 1.02 (0.63–1.67) | 0.925 | 0% | 0.73 |

**Abbreviations:** K: number of studies. N: number of participants. OR: odds ratio; CI: Confidence interval; NOACs: non-vitamin K antagonist oral anticoagulants; VKAs: vitamin K antagonists; SSE: stroke or systemic embolism.

**Meta-analysis based on generalized linear mixed-model**


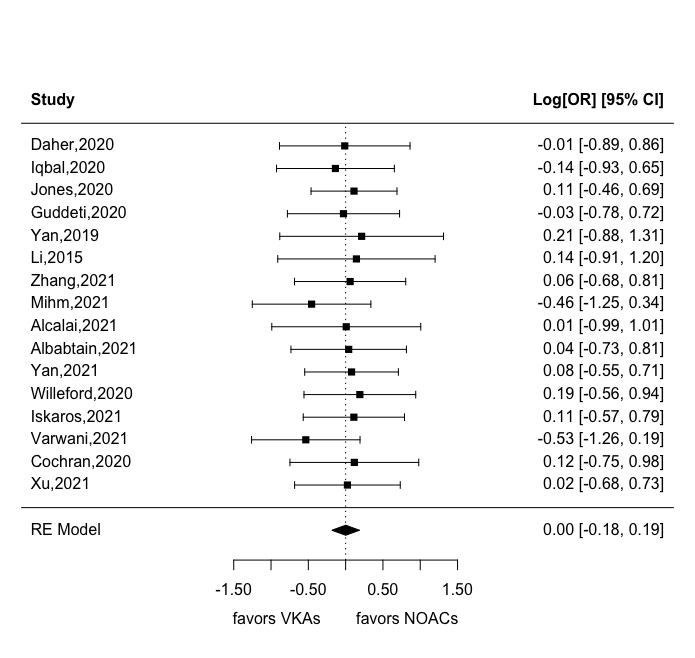


**Supplementary Fig. 1.** **Forest plot of thrombus resolution (NOACs versus VKAs) based on the generalized linear mixed-model.** Abbreviation: OR: odds ratio; CI: Confidence interval; NOACs: non-vitamin K antagonist oral anticoagulants; VKAs: vitamin K antagonists.


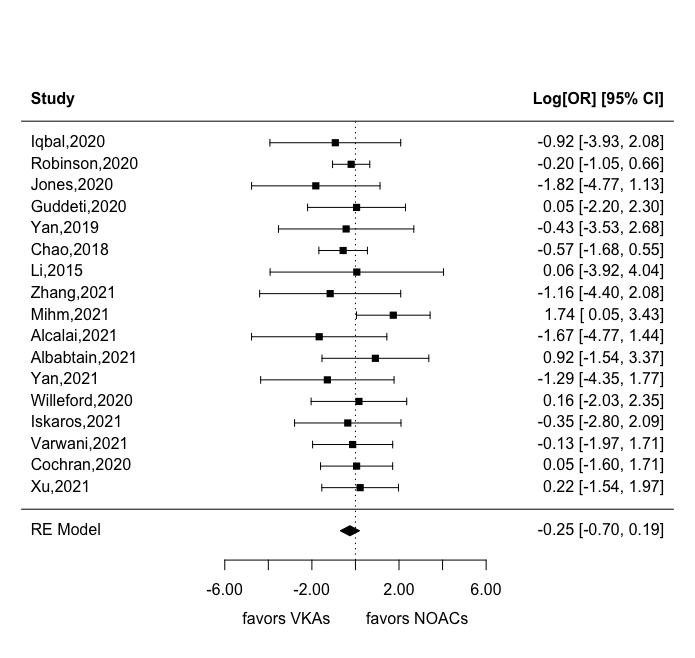


**Supplementary Fig. 2.** **Forest plot of bleeding events (NOACs versus VKAs) based on the generalized linear mixed-model.** Abbreviation: OR: odds ratio; CI: Confidence interval; NOACs: non-vitamin K antagonist oral anticoagulants; VKAs: vitamin K antagonists.


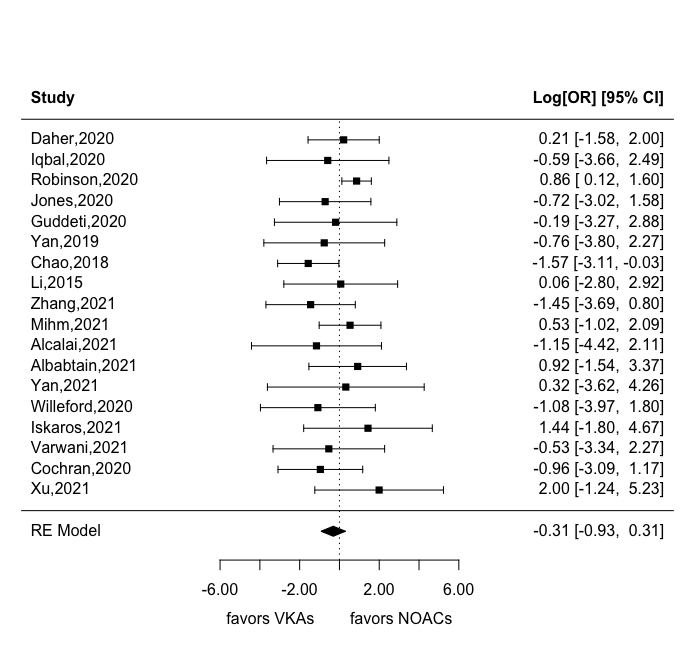


**Supplementary Fig. 3.** **Forest plot of stroke or systemic embolism events (NOACs versus VKAs) based on the generalized linear mixed-model.** Abbreviation: OR: odds ratio; CI: Confidence interval; NOACs: non-vitamin K antagonist oral anticoagulants; VKAs: vitamin K antagonists.


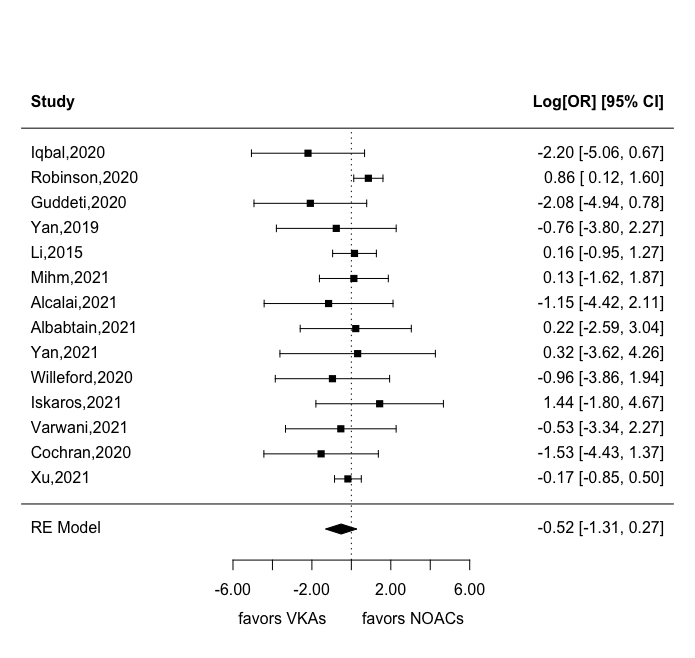


**Supplementary Fig. 4.** **Forest plot of stroke (NOACs versus VKAs) based on the generalized linear mixed-model.** Abbreviation: OR: odds ratio; CI: Confidence interval; NOACs: non-vitamin K antagonist oral anticoagulants; VKAs: vitamin K antagonists.


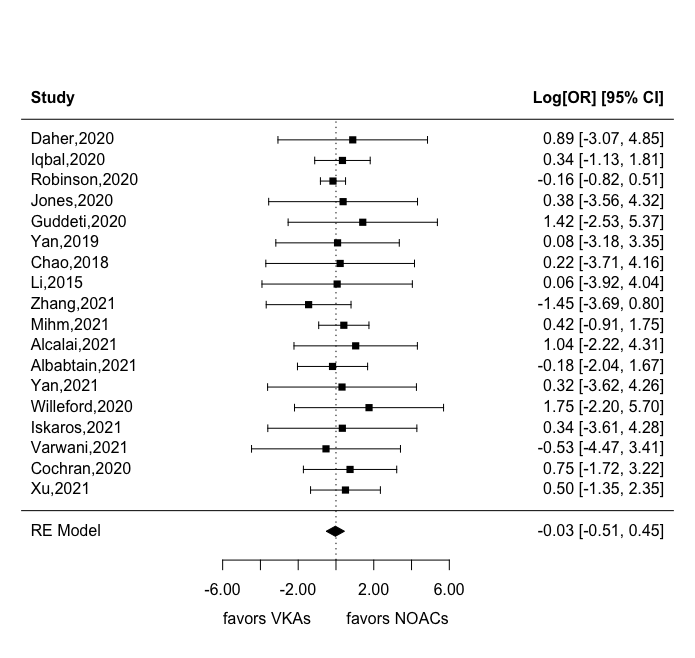


**Supplementary Fig. 5.** **Forest plot of all-cause of death (NOACs versus VKAs) based on the generalized linear mixed-model.** Abbreviation: OR: odds ratio; CI: Confidence interval; NOACs: non-vitamin K antagonist oral anticoagulants; VKAs: vitamin K antagonists.

**Subgroup analyses**


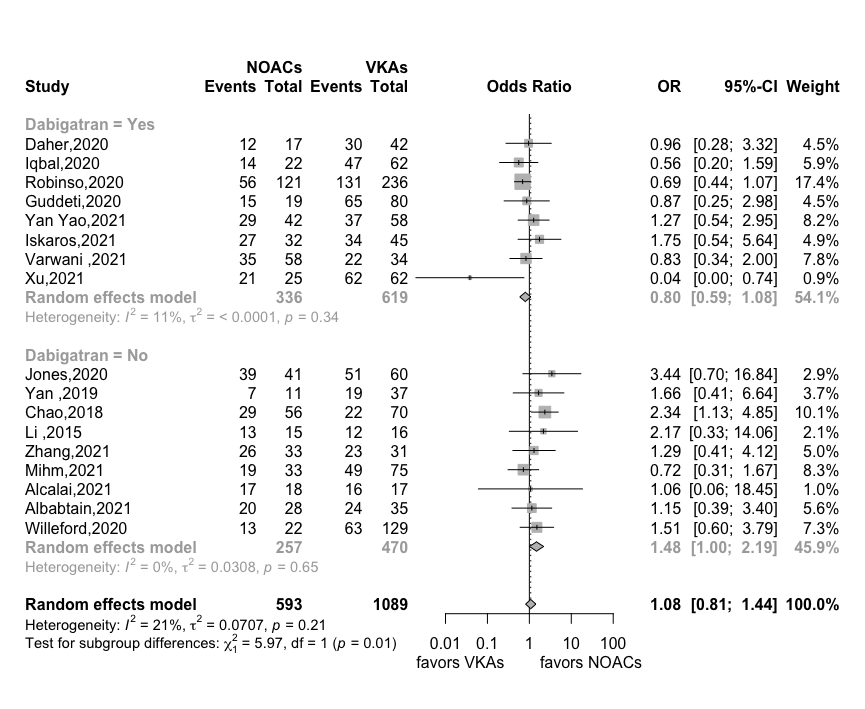


**Supplementary Fig. 6.** **Subgroup analyses on the thrombus resolution (NOACs versus VKAs).** The subgroup was divided as studies in which the NOACs groups use included dabigatran (Yes) or not included dabigatran (No). Abbreviation: OR: odds ratio; CI: Confidence interval; NOACs: non-vitamin K antagonist oral anticoagulants; VKAs: vitamin K antagonists.


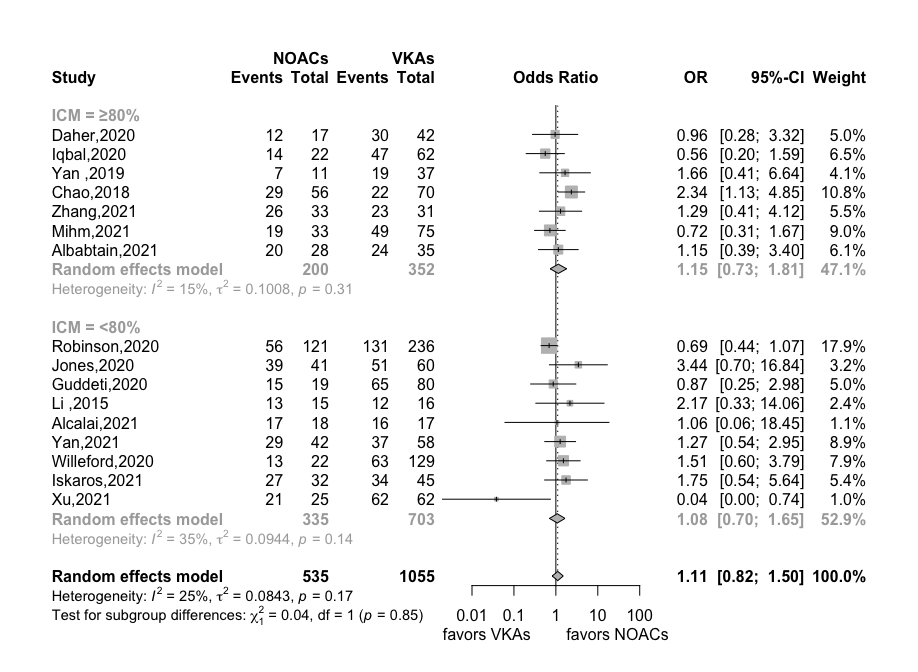


**Supplementary Fig. 7.** **Subgroup analyses on the thrombus resolution (NOACs versus VKAs).** The subgroup was divided as a very high rate of ICM history (≥80%) and a moderate high or low rate (<80%). Abbreviation: OR: odds ratio; CI: Confidence interval; NOACs: non-vitamin K antagonist oral anticoagulants; VKAs: vitamin K antagonists; ICM: ischemic cardiomyopathy.


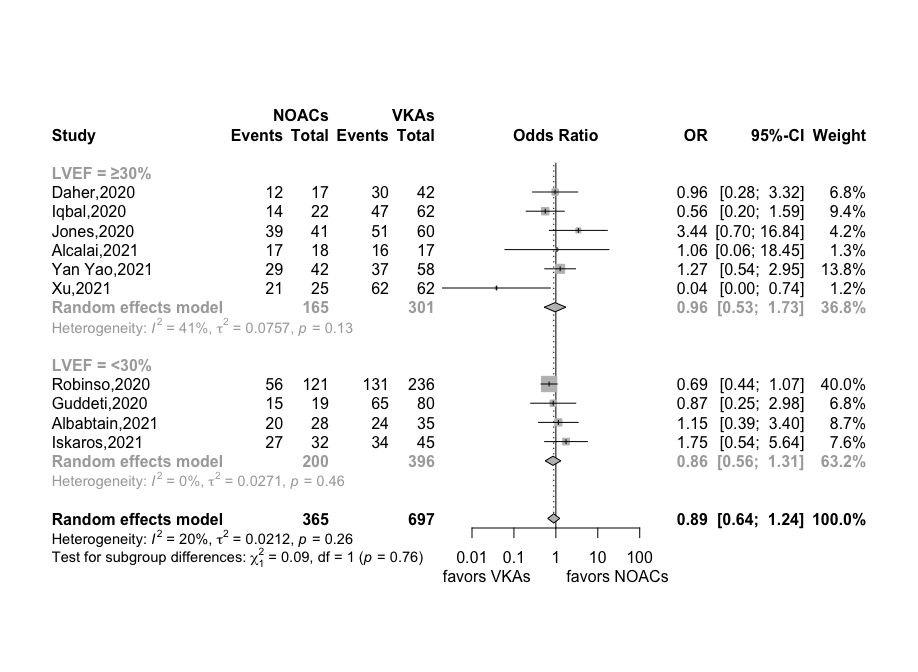


**Supplementary Fig. 8.** **Subgroup analyses on the thrombus resolution (NOACs versus VKAs).** The subgroup was divided as a moderate low LVEF (≥30%) and a very low LVEF (<30%). Abbreviation: OR: odds ratio; CI: Confidence interval; NOACs: non-vitamin K antagonist oral anticoagulants; VKAs: vitamin K antagonists; LVEF: left ventricular ejection fraction.


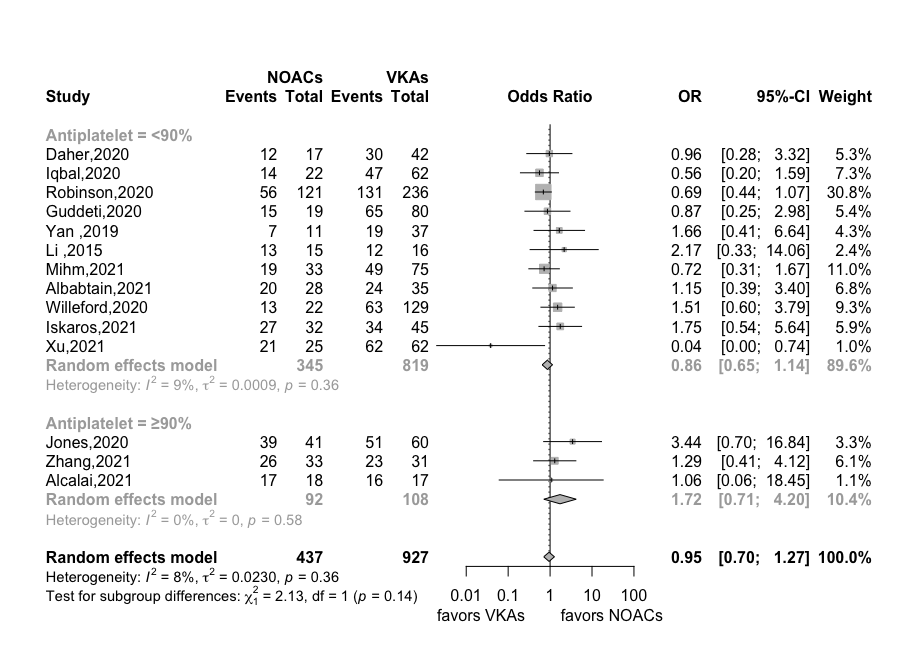


**Supplementary Fig. 9.** **Subgroup analyses on the thrombus resolution (NOACs versus VKAs).** The subgroup was divided as a moderate high or low rate (<90%) and a very high rate of combination with antiplatelet therapy (≥90%). Abbreviation: OR: odds ratio; CI: Confidence interval; NOACs: non-vitamin K antagonist oral anticoagulants; VKAs: vitamin K antagonists.

**Sensitivity analysis**


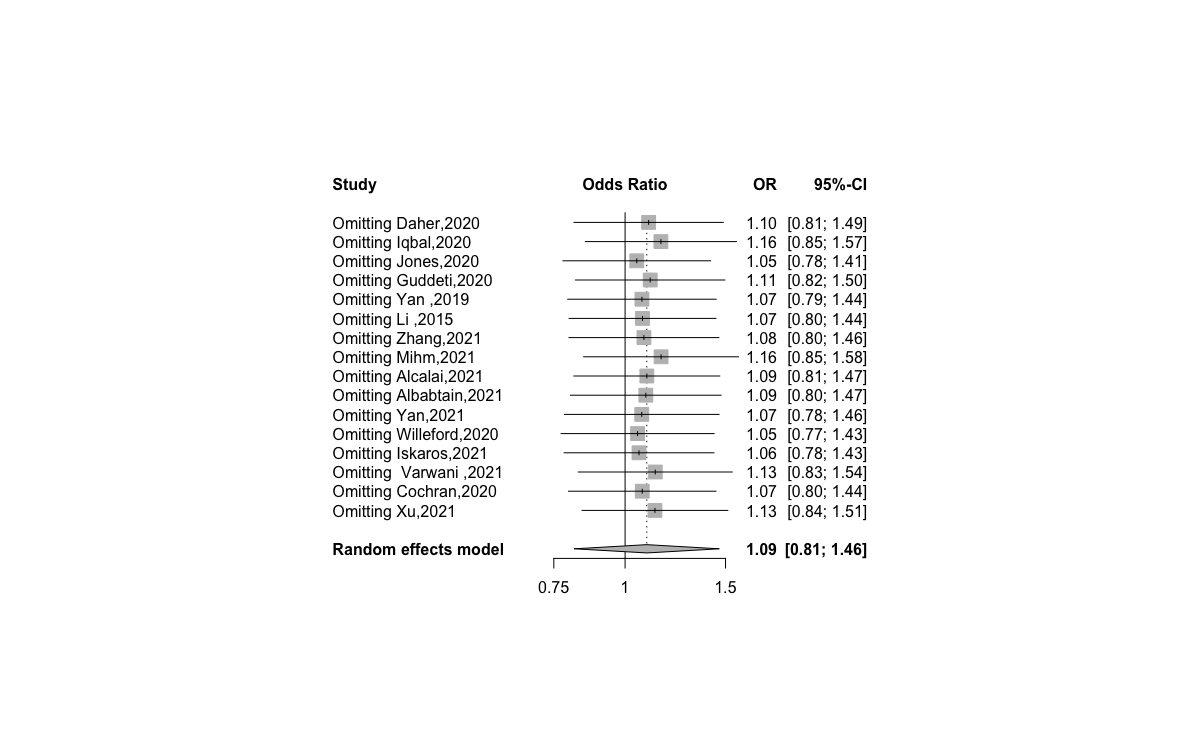


**Supplementary Fig. 10. Sensitivity analysis on thrombus resolution (16 studies).** Abbreviation: OR: odds ratio; CI: Confidence interval.


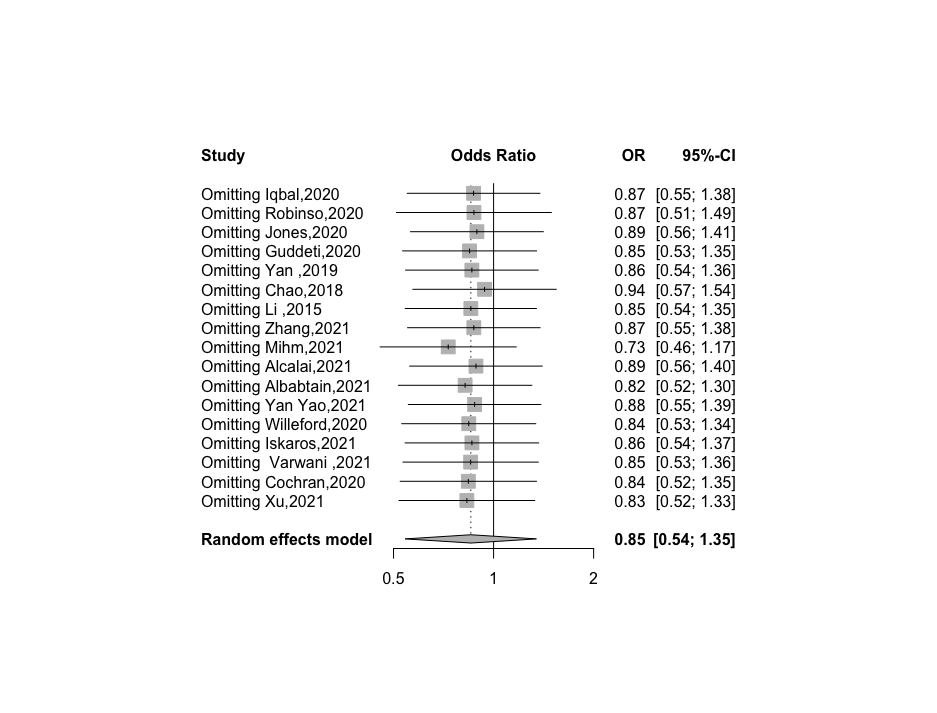


**Supplementary Fig. 11. Sensitivity analysis on bleeding (17 studies).** Abbreviation: OR: odds ratio; CI: Confidence interval.


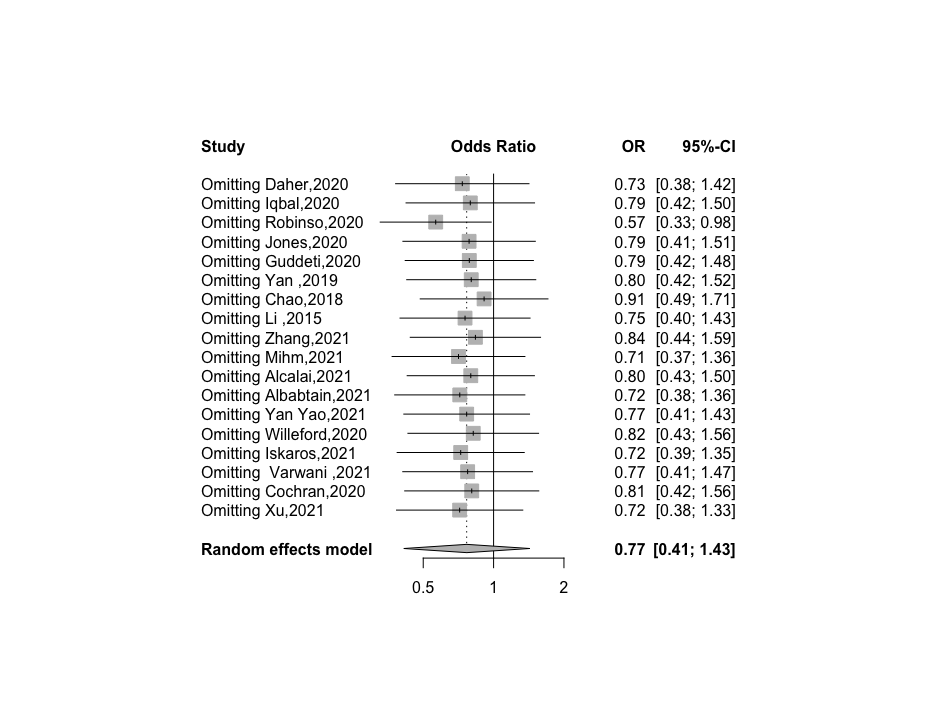


**Supplementary Fig. 12. Sensitivity analysis on stroke or systemic embolism (18 studies).** Abbreviation: OR: odds ratio; CI: Confidence interval.


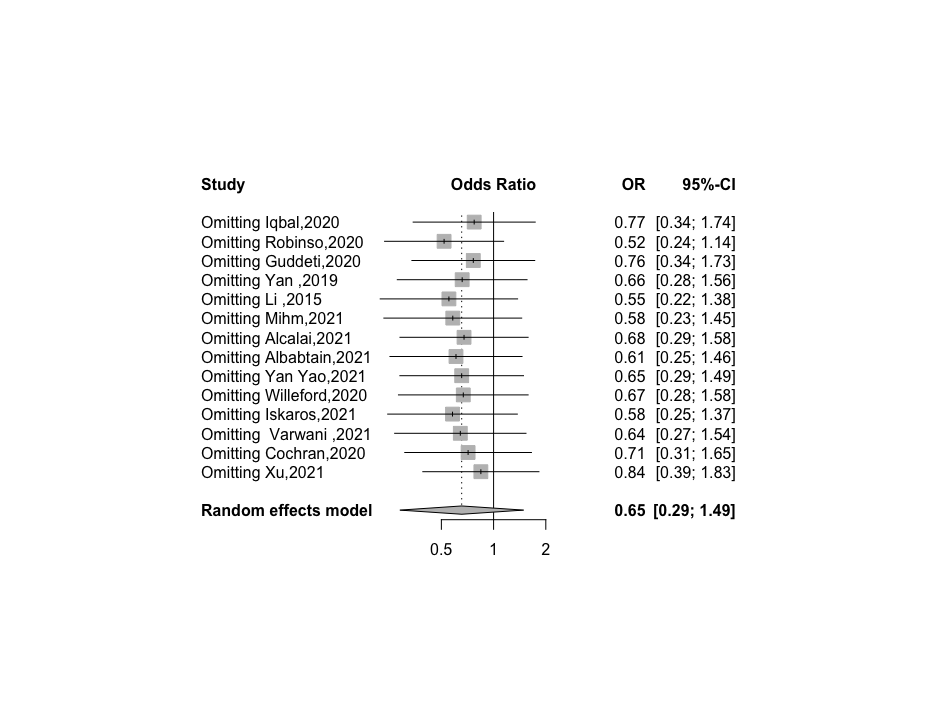


**Supplementary Fig. 13. Sensitivity analysis on stroke (14 studies).** Abbreviation: OR: odds ratio; CI: Confidence interval.


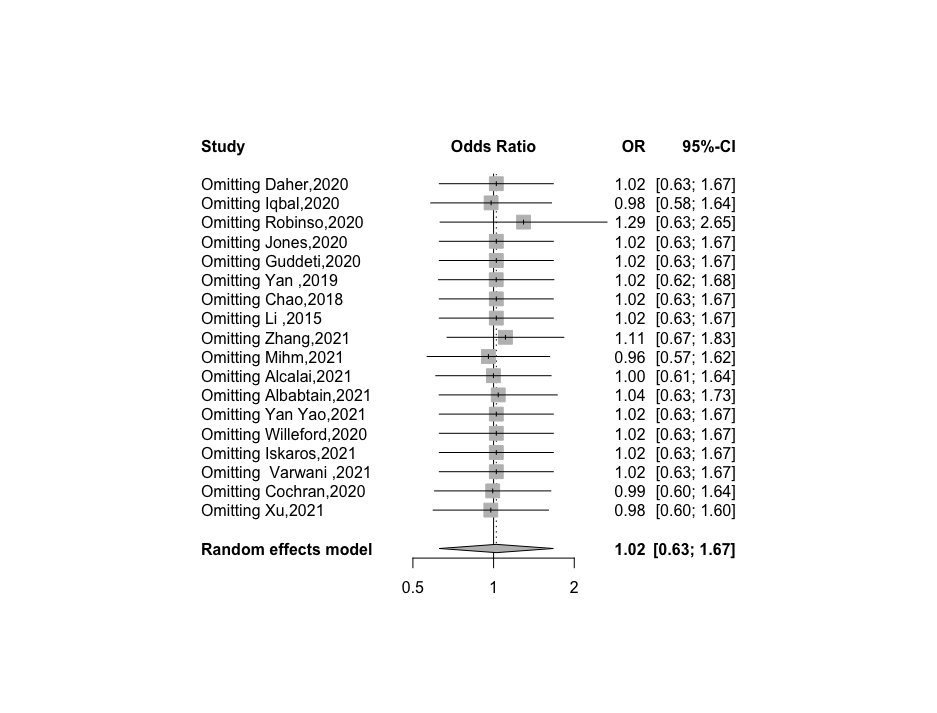


**Supplementary Fig. 14. Sensitivity analysis on all-cause death (18 studies).** Abbreviation: OR: odds ratio; CI: Confidence interval.

**Publication bias**


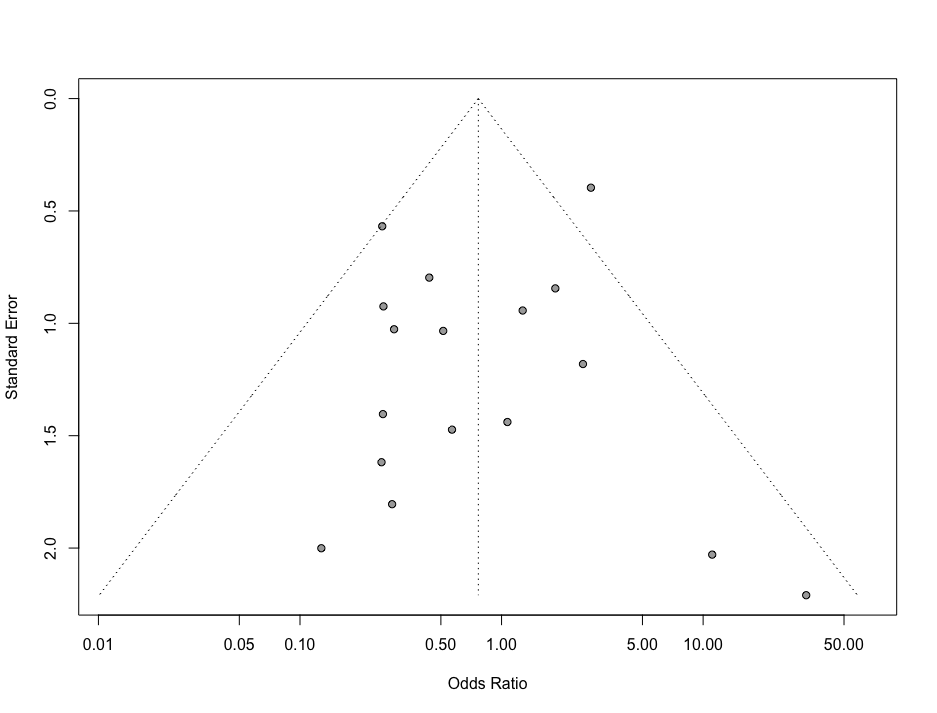


**Supplementary Fig. 15. Funnel plot for publication bias with 18 eligible studies.**


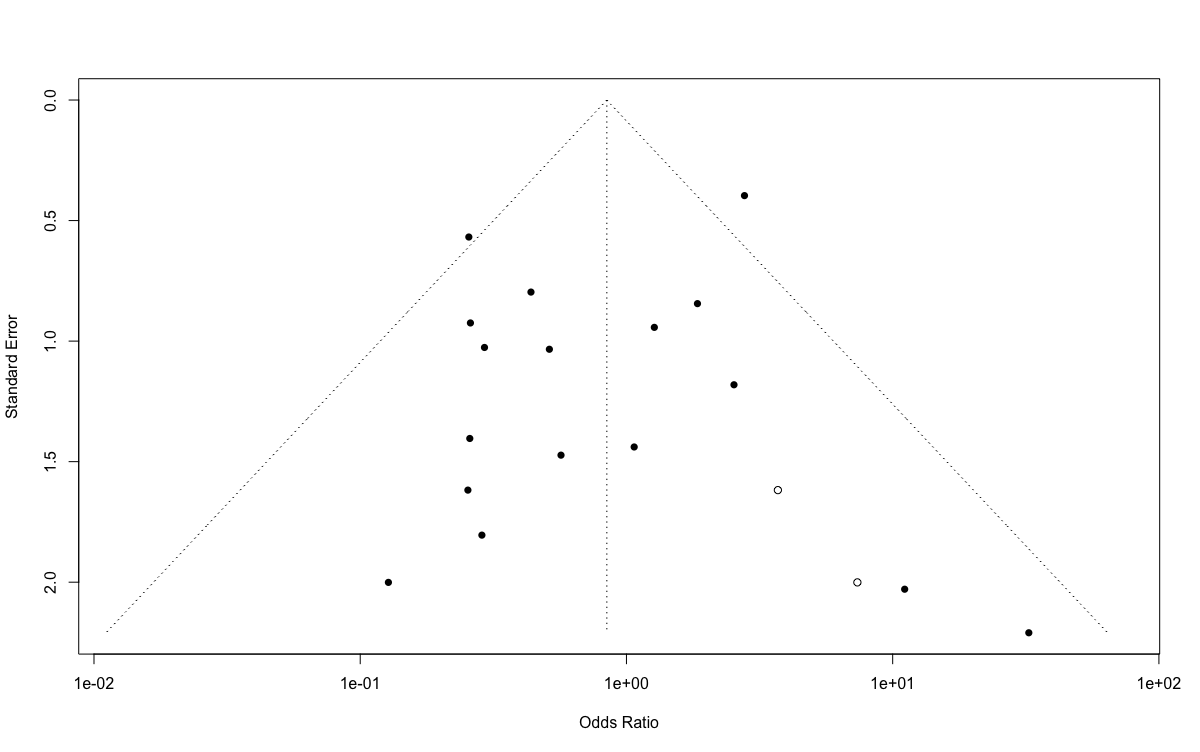


**Supplementary Fig. 16. Funnel plot after trimmed with 18 eligible studies.**

Search Strategy

**Pubmed/Medline**

((((“Ventricular Thromb*”[Mesh] OR“Apical Thromb*”[Mesh] OR “Intraventricular Thromb*”[Mesh] OR “Left Ventricular Thromb*”[Mesh] OR “Right Ventricular Thromb*”[Mesh] OR “ventricular thromb*” OR“apical thromb*” OR “intraventricular thromb* “ OR “left ventricular thromb*” OR “right ventricular thromb*”))) AND((“Anticoagulants”[Mesh] OR “Antithrombins”[Mesh] OR “Factor Xa Inhibitors”[Mesh] OR “blood clotting factor 10a inhibitor” OR “dabigatran” OR “dabigatran etexilate” OR “rivaroxaban” OR “apixaban” OR “edoxaban” OR pradaxa OR pradax OR pradaxar OR prazaxa OR rendix OR xarelto OR eliquis OR savaysa OR lixiana OR “target-specific oral anticoagulants” OR “target specific oral anticoagulants” OR tsoacs OR tsoac OR “new oral anticoagulants” OR “new oral anticoagulant” OR “novel oral anticoagulants” OR “novel oral anticoagulant” OR noacs OR noac OR “direct oral anticoagulants” OR “direct oral anticoagulant” OR doacs OR doac OR “antithrombin” OR “anticoagulant” OR “anticoagulants” OR “factor xa inhibitor”))) AND((“Warfarin”[Mesh] OR “Coumarins”[Mesh] OR “Acenocoumarol”[Mesh] OR “Phenprocoumon”[Mesh] OR “Phenindione”[Mesh] OR “warfarin” OR “coumadin” OR “courmarin anticoagulant” OR “vitamin k antagonist” OR “antivitamin k” OR “phenprocoumon” OR “phenprocumon” OR “acenocumarol” OR “acenocoumarol” OR “fluindione” OR “phenindione” OR “anisindione”))

**Embase**

#1 'ventricular thromb$' OR 'left ventricular thromb$'/exp OR 'right ventricular thromb$' OR 'ventricular thromb$' OR 'left ventricular thromb$' OR 'right ventricular thromb$’

AND

#2 ‘anticoagulant agent’/exp OR ‘anticoagulation’/exp OR ‘blood clotting factor 10a inhibitor’/exp OR ‘antithrombin’/exp OR ‘dabigatran’/exp OR ‘dabigatran etexilate’/exp OR ‘rivaroxaban’/exp OR ‘apixaban’/exp OR ‘edoxaban’/exp OR dabigatran OR rivaroxaban OR apixaban OR edoxaban OR pradaxa OR pradax OR pradaxar OR prazaxa OR rendix OR xarelto OR eliquis OR savaysa OR lixiana OR ‘target-specific oral anticoagulants’ OR ‘target specific oral anticoagulants’ OR tsoacs OR tsoac OR ‘new oral anticoagulants’ OR ‘new oral anticoagulant’ OR ‘novel oral anticoagulants’ OR ‘novel oral anticoagulant’ OR noacs OR noac OR ‘direct oral anticoagulants’ OR ‘direct oral anticoagulant’ OR doacs OR doac OR ‘antithrombin’ OR ‘anticoagulant’ OR ‘anticoagulants’ OR ‘factor xa inhibitor’

AND

#3 ‘coumarin anticoagulant’/exp OR ‘warfarin’/exp OR ‘antivitamin k’/exp OR warfarin OR coumadin OR ‘courmarin anticoagulant’ OR ‘vitamin k antagonist’ OR ‘antivitamin k’ OR phenprocoumon OR phenprocumon OR acenocumarol OR acenocoumarol OR fluindione OR phenindione OR anisindione

**Web of Science**

TS=(‘ventricular thromb*’ OR ‘left ventricular thromb*’ OR ‘right ventricular thromb*’ OR ‘apical thromb*’ OR ‘intraventricular thromb*’)

AND

TS=(‘blood clotting factor 10a inhibitor’ OR ‘dabigatran’ OR ‘dabigatran etexilate’ OR ‘rivaroxaban’ OR ‘apixaban’ OR ‘edoxaban’ OR pradaxa OR pradax OR pradaxar OR prazaxa OR rendix OR xarelto OR eliquis OR savaysa OR lixiana OR ‘target-specific oral anticoagulants’ OR ‘target specific oral anticoagulants’ OR tsoacs OR tsoac OR ‘new oral anticoagulants’ OR ‘new oral anticoagulant’ OR ‘novel oral anticoagulants’ OR ‘novel oral anticoagulant’ OR noacs OR noac OR ‘direct oral anticoagulants’ OR ‘direct oral anticoagulant’ OR doacs OR doac OR ‘antithrombin’ OR ‘anticoagulant’ OR ‘anticoagulants’ OR ‘factor xa inhibitor’)

AND

TS=(warfarin OR coumadin OR ‘courmarin anticoagulant’ OR ‘vitamin k antagonist’ OR ‘antivitamin k’ OR phenprocoumon OR phenprocumon OR acenocumarol OR acenocoumarol OR fluindione OR phenindione OR anisindione)

**Cochrane Library**

#1 MeSH descriptor:[Ventricular Thromb*] explode all trees

#2 MeSH descriptor:[Left Ventricular Thromb*] explode all trees

#3 MeSH descriptor:[Right Ventricular Thromb*] explode all trees

#4 MeSH descriptor:[Apical Thromb*] explode all trees

#5 MeSH descriptor:[Intraventricular Thromb*] explode all trees

#6 #1 or #2 or #3 or or #4 or #5 or ‘ventricular thromb*’or ‘left ventricular thromb*’or ‘right ventricular thromb*’ or ‘apical thromb*’ or ‘intraventricular thromb*’

#7 MeSH descriptor:[Blood Coagulation Factor Inhibitors] explode all trees

#8 MeSH descriptor:[Anticoagulants] explode all trees

#9 MeSH descriptor:[Antithrombins] explode all trees

#10 MeSH descriptor:[Factor Xa Inhibitors] explode all trees

#11 #7 or #8 or #9 or #10 or ‘blood clotting factor 10a inhibitor’ or ‘dabigatran’ or ‘dabigatran etexilate’ or ‘rivaroxaban’ or ‘apixaban’ or ‘edoxaban’ or pradaxa or pradax or pradaxar or prazaxa or rendix or xarelto or eliquis or savaysa or lixiana or ‘target-specific oral anticoagulants’ or ‘target specific oral anticoagulants’ or tsoacs or tsoac or ‘new oral anticoagulants’ or ‘new oral anticoagulant’ or ‘novel oral anticoagulants’ or ‘novel oral anticoagulant’ or noacs or noac or ‘direct oral anticoagulants’ or ‘direct oral anticoagulant’ or doacs or doac or ‘antithrombin’ or ‘anticoagulant’ or ‘anticoagulants’ or ‘factor xa inhibitor’

#12 MeSH descriptor:[Warfarin] explode all trees

#13 MeSH descriptor:[Coumarins] explode all trees

#14 MeSH descriptor:[Acenocoumarol] explode all trees

#15 MeSH descriptor:[Phenprocoumon] explode all trees

#16 MeSH descriptor:[Phenindione] explode all trees

#17 #12 OR #13 OR #14 OR #15 OR #16 OR warfarin OR coumadin OR ‘courmarin anticoagulant’ OR ‘vitamin k antagonist’ OR ‘antivitamin k’ OR phenprocoumon OR phenprocumon OR acenocumarol OR acenocoumarol OR fluindione OR phenindione OR anisindione

#18 #6 AND #11 AND #17
